# Towards valid and reusable reference alignments — ten basic quality checks for ontology alignments and their application to three different reference data sets

**DOI:** 10.1186/2041-1480-3-S1-S4

**Published:** 2012-04-24

**Authors:** Elena Beisswanger, Udo Hahn

**Affiliations:** 1 Jena University Language and Information Engineering (JULIE) Lab, Friedrich-Schiller-Universität Jena, Jena, Germany

## Abstract

Identifying relationships between hitherto unrelated entities in different ontologies is the key task of ontology alignment. An alignment is either manually created by domain experts or automatically by an alignment system. In recent years, several alignment systems have been made available, each using its own set of methods for relation detection. To evaluate and compare these systems, typically a manually created alignment is used, the so-called reference alignment. Based on our experience with several of these reference alignments we derived requirements and translated them into simple quality checks to ensure the alignments’ validity and also their reusability. In this article, these quality checks are applied to a standard reference alignment in the biomedical domain, the Ontology Alignment Evaluation Initiative Anatomy track reference alignment, and two more recent data sets covering multiple domains, including but not restricted to anatomy and biology.

## Background

In knowledge-intensive domains such as the life sciences, there is an ever-increasing need for concept systems and ontologies to organize and classify the large amounts of clinical and lab data and to describe such data collections with value-adding meta data. For this purpose, numerous ontologies with different levels of coverage, expressiveness and formal rigor have evolved that, from a content point of view, complement each other and in some cases even overlap. To facilitate the interoperability between information systems using different ontologies and to detect overlaps between them, ontology alignment has become a crucial need.

Since the manual alignment of ontologies is quite labor-expensive and time-consuming, alignment tools have been developed that can automatically detect correspondences between entities in different ontologies. Following Euzenat and Shvaiko [1], we consider entities to comprise ontology classes, class instances, and properties, the latter corresponding to roles in the terminology of description logics. A correspondence consists of a pair of entities (e.g., a class from the first input ontology, O_1_, and a class from the second one, O_2_) and a relation that, according to the creator of the alignment, holds between these entities (e.g., an *equivalentClass* or *subClassOf* relation between two classes).

Many different approaches to and techniques for ontology alignment have been proposed up until now (see, e.g., [2-7]), and dedicated scientific workshops have been organized to accelerate the progress in this field. In 2005, the Ontology Alignment Evaluation Initiative (OAEI) [8] initiated a series of annual evaluation events to monitor and compare the quality of different alignment systems. A somewhat broader view on the evaluation of semantic technologies is promoted by the Semantic Evaluation At Large Scale (SEALS) project [9] that started in 2009. An open source platform is under development to facilitate the remote evaluation of ontology alignment systems and other semantic technologies in terms of both, large-scale evaluation campaigns but also *ad hoc* evaluations of single systems. Amongst others, the platform provides a test data repository, a tools repository, and a results repository for the evaluation and comparison of systems.

The most valuable contents of the SEALS platform’s test data repository and also the core of the OAEI campaigns are manually created or at least manually curated reference alignments which constitute the ground truth against which alignment systems are to be evaluated. Clearly, the quality of these reference alignments is of paramount importance for the validity of the evaluation results. For the evaluation of our own ontology alignment system, we were also looking for valid test data (ontologies and reference alignments). Some data sets we inspected have been used for several years in the OAEI campaigns, or have already been integrated in the SEALS test data repository. Others have just recently been published and have not been used in any public challenge up until now. Notwithstanding the enormous efforts that have gone into the development of such resources, our inspection of many different data sets revealed a number of content-specific shortcomings and technical deficiencies. Hence, we decided to formulate a list of basic quality checks which summarize these observations. We propose to apply these checks to any given alignment as a kind of minimal validity test before it is used as a reference standard in any evaluation.

In the remainder of this paper, we will first introduce the basic requirements we have defined and then we will apply them to one of the standard data sets used in the yearly OAEI campaigns, the Anatomy track reference alignment, and two quite recent alignment data sets that we think are of interest since they cover various different domains and hold correspondences based on (strict) *subClassOf* relations, in addition to the much more common *equivalentClass*-based correspondences. Finally, we will discuss how the application of the checks to these data sets can lead to an improved version of both, the reference alignments themselves and some of the input ontologies.

## Basic quality checks for reference alignments

**Figure 1:**
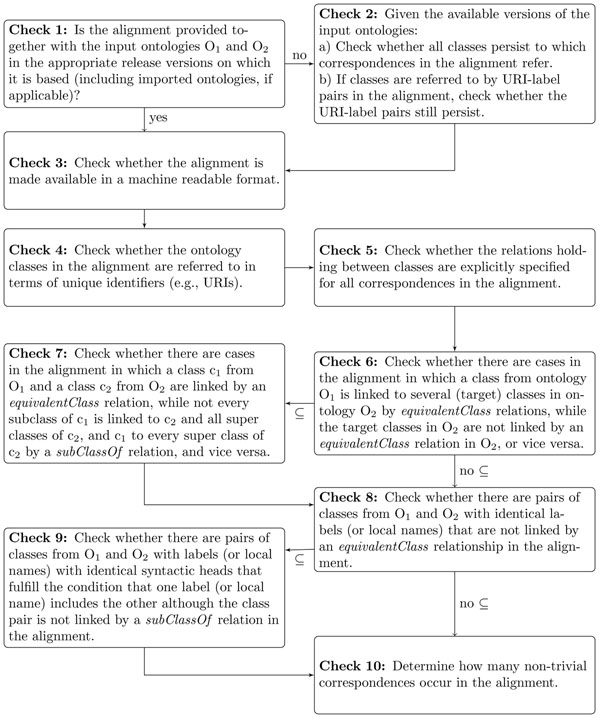
**Ten basic quality checks for ontology alignments.** Ten basic quality checks for ontology alignments and the proposed order of execution. Concerning Check 6 and Check 8, if the alignment on which the checks are made are supposed to incorporate *subClassOf*-based correspondences, follow the arrows marked with “⊆”, otherwise those with “no ⊆”.

An alignment consists of a set of correspondences between entities from two different ontologies. In our current work, we focus on *equivalentClass* and *subClassOf*-based correspondences between ontology classes only. These are the two most popular types of correspondences considered in the ontology alignment community. The usefulness of a manually created or curated alignment as reference for the evaluation of ontology alignment systems depends on various parameters. The quality checks presented in Figure 1 address fundamental validity and reusability concerns.

The first five quality checks focus on the (re)usability of an alignment as reference for the evaluation of alignment systems. Checks 1 and 2a) test whether the correspondences contained in the alignment can be found at all by the alignment systems based on the available release versions of the input ontologies (imagine the case where a class has been deleted from an input ontology—consequently, correspondences in the reference alignment referring to this class cannot be reproduced anymore). Check 2b), which tests for label (i.e., class name) changes, is targeted at the tacit evolution of the meaning of a class. In particular for light-weight ontologies lacking thorough formal class definitions, verbal labels virtually carry the entire meaning of a class and, hence, a new label might indicate a subtle or even severe change of the meaning of an ontology class requiring further scrutiny. If Check 1 is positive, Check 2 can be skipped. Check 3 is concerned with the accessibility of an alignment, while Check 4 aims at finding out whether the references to classes are unique. For example, imagine the case where local names or labels would be given as class references, then those references might be ambiguous. Note that “local name” refers to the terminal part of a URI. In many RDF(S)/OWL ontologies, especially if they do not provide explicit class labels, the name of a class is encoded in its local name, such as “anatomical structure” in “http://dbpedia.org/ontology/AnatomicalStructure” Check 5 is meant to assure that explicit semantic types are specified for the relationships asserted between the classes by the alignment creators.

The remaining five checks address the completeness (Checks 6 to 9) and the non-trivialness of the alignment (Check 10). While Checks 6 and 7 work on the structural level exploiting the class hierarchy of the input ontologies to find evidences for possibly missing or erroneous correspondences, Checks 8 and 9 address the same concern but target at the language level instead, exploiting class labels. Since in an alignment a class from one ontology should be mapped to at most one class in the other ontology by an *equivalentClass* relation (or, if it links to several classes, these should be marked as being equivalent themselves), Check 6 may provide valuable hints for redundant or even mistaken correspondences in an alignment, but also for implicit class equivalences in the input ontologies. Check 7 exploits the fact that stating an *equivalentClass* relation between two classes logically entails *subClassOf* relations between all subclasses of one class and all super classes of the other. We may thus identify missing *sub ClassOf*-based correspondences, but may also detect hints to erroneous *equivalentClass-*based correspondences in the alignment or modeling errors in the input ontologies. Checks 8 and 9 reflect the observation that when two ontologies are aligned, especially when they show a strong conceptual overlap, label identity between classes provides strong evidence for class equivalence, whereas labels with identical syntactic heads that fulfill the condition that one label includes the other are a strong indicator for concept subsumption. Examples include the label pairs “*doctoral* thesis” and “thesis”, and “professor *of biology*” and “professor” (with rightmost and leftmost heads, respectively). Both checks may help in detecting missing correspondences in an existing alignment. Checks 7 and 9 may be skipped when *subClassOf*-based correspondences are out of the scope of the alignment under scrutiny. Finally, Check 10 allows for a stricter evaluation of the capabilities of an ontology alignment system by distinguishing between relaxed and tight test conditions. In the relaxed mode, the determination of lots of trivial correspondences may overestimate the true potential of an alignment system, simply because for finding trivial correspondences exact (sub)string matching is entirely sufficient. As “trivial” we define *equivalentClass*-based correspondences that can be detected via the identity of class labels (or local names), and *subClassOf*-based correspondences that can be detected via mere syntactic head analysis of class labels (or local names), both after applying a simple term normalization procedure. In the strict mode, however, only non-trivial correspondences are taken into account rendering evidence for the true sophistication of the alignment finding procedure. Certainly, a large proportion of trivial correspondences in an alignment (an indication of strongly overlapping input ontologies) decreases its value as reference alignment, although trivial correspondences do play a certain role as anchors for advanced alignment strategies [2].

## Sample data sets

To illustrate the power of the proposed quality checks we apply them to three different data sets (see Table 1). The first one (referred to as ANATOMY) is a standard reference alignment in the biomedical domain that has been used in the Anatomy track of the OAEI campaign since 2007. The second and third one (referred to as LOD, and BRIDGE, respectively) are two more recent data sets, one created for evaluating alignments of Linked Open Data schemas, the other for assessing upper ontology-based alignment approaches. Both excel in a broad domain coverage and in the provision of *equivalentClass* and *subClassOf*-based correspondences. Yet, two of the data sets have complementarily balanced correspondences, ANATOMY favoring *equivalentClass* relations, LOD featuring *subClassOf* relations. According to the authors of the three data sets all correspondences concern pairs of ontology classes.

**Table 1:** Overview on the Anatomy, Lod and Bridge data sets.

Data set	Domain	Alignments	Ontologies	≡	⊆	All corresp.
ANATOMY	Anatomy	1	2	1,520	0	**1,520**
LOD	Various	7	8	85	2,260	**2,339**
BRIDGE	Various	10	17	Unknown	Unknown	**4,876**

Overview on the ANATOMY, LOD and BRIDGE data sets. The symbols ≡ and ⊆ stand for *equivalentClass* and *subClassOf*-based correspondences, respectively.

### OAEI anatomy track reference alignment

The original OAEI Anatomy track reference alignment links pairs of equivalent classes from the anatomy branch of the NCI Thesaurus (NCI) [10], describing human anatomy, and the mouse adult gross anatomy ontology (MA) based on the Anatomical Dictionary for the Adult Mouse [11]. This alignment was created in a combined manual and automatic effort — the automatic alignment, exploiting lexical and structural techniques, was followed by an extensive manual curation step [12]. In the OAEI 2010 Anatomy track, a slightly revised version of the original alignment was used that contains 1,520 *equivalentClass*-correspondences.

### Reference alignments for linked open data schemas

To evaluate an alignment system tuned to cross-link schemas of Linked Open Data (LOD) sets, Jain et al. [5] manually created seven reference alignments between selected pairs of eight such schemas, covering general information as well as particular domains ranging from geography over scientific publications to entertainment and social networks. The reference alignments comprise a total of 2,339 distinct correspondences, the vast majority of which (over 96%) relating to *subClassOf* relations, the remaining ones to *equivalentClass* relations.

### Reference alignments for testing upper ontology-based alignment approaches

Another multi-domain data set has been created by Mascardi et al. [4] to evaluate structural alignment approaches exploiting upper ontologies as semantic bridges in the alignment process. The data set consists of ten manually created reference alignments between selected pairs of 17 input ontologies covering various domains, ranging from anatomy, biology and geography to gastronomy, travel and entertainment. The reference alignments contain a total of 4,876 distinct correspondences based on three different relation types: *equivalentClass*, *subClassOf*, and a generic *relatedTo* relation. However, according to the authors of the data set, in the evaluation for which the alignments have been created, no distinction was made between the three relation types so that Table 1 lacks coverage statistics for *equivalentClass* and *subClassOf* correspondences.

## Results of applying the quality checks

We ran the ten basic quality checks on the data sets introduced above and achieved the following results:

### Check 1

In the ANATOMY data set, the reference alignment is used together with a version of the NCI Thesaurus anatomy branch as from 2006-02-13, and a version of the MA as from 2007-01-18 (both in OWL format), while the alignment itself was created based on the NCI Thesaurus release version 04.09a (from 2004-09-10) and the MA version as from 2004-11-22 [12]. Obviously, different release versions of the input ontologies have been mixed up for the creation of the reference alignment and for running the OAEI Anatomy track. In the LOD and the BRIDGE data sets no input ontologies are included at all. Instead, in the respective publications URLs for download are provided [4,5].

When we tried to download the ontologies from the specified URLs, we encountered two major problems with that approach. First, we observed that many ontology URLs do not point to a distinct ontology version. Instead, they either specify a webspace at which always the most recent version of the respective ontology is available, or they refer to a general website of the ontology that provides download links for both, the most recent but also older versions of the ontology. While in the first case, the user cannot choose among alternative (in particular, older) ontology versions anymore at the specified URL, in the second case the user misses the information which version is the required one. We decided to download always the most recent ontology version. The second problem emerges from the fact that the Web is not static at all. Contents can be moved or deleted, resulting in broken URLs. This happed already to 8 of the 17 URLs specified as sources for input ontologies of the BRIDGE alignments and, additionally, to a number of ontologies imported by them. Six of the “vanished” ontologies we were able to retrieve from the cache of the semantic web search engine Swoogle [13], two we could find searching the Web. So, at least we were able to proceed and run the remaining quality checks.

### Check 2a)

Check 1 revealed that we were working with input ontology versions different from those the respective alignments were based on (or, at least, we cannot guarantee that we were dealing with the correct versions). Thus this check was compulsory for all three data sets. For the ANATOMY data set we found that all classes involved in the alignment (i.e., participating in at least one correspondence), are still contained in the available versions of the input ontologies. Hence, class consistency is preserved. For the LOD alignments we found a total of 143 classes missing in the downloaded input ontologies affecting 413 correspondences, and for the BRIDGE alignments 12 classes had disappeared affecting 18 correspondences.

However, analyzing the set of “vanished” correspondences in the LOD and the BRIDGE data set we discovered that actually much fewer classes were missing than one might have thought considering the results from Check 2a). In fact, in many cases simply errors in the alignment files (such as ordinary character errors or omissions, or mixed up name spaces) precluded the recovery of classes in the input ontologies. Just as an example, when we removed erroneous whitespace from local names of classes in the LOD alignment mapping tables, the number of classes referred to in the alignments but not available in the corresponding input ontologies dropped from 143 to the much smaller number of 47, and the number of vanished correspondences decreased from 413 to 182. (Because of the strong impact of whitespace removal we took the revised versions of the LOD alignments as basis for all further checks.) As another reason for only *seemingly* missing classes we found that in rare cases the BRIDGE alignments refer to ontology elements that are not explicitly specified as classes in the input ontologies (i.e., they are not typed as owl:Class or rdfs:Class, a requirement that we included in our implementation of the quality checks) and thus were not found.

### Check 2b)

Although in the ANATOMY alignment classes involved in correspondences were specified by URIs, we received from a curator of the alignment the original mapping table on which the alignment was based. The mapping table lists both, URIs as well as the labels of class pairs. We tested whether the URI-class label combinations are still valid in the new versions of the input ontologies and found 85 NCI classes and 34 MA classes for which the labels had changed. A manual inspection revealed that in most cases labels had been made more precise in the new ontology versions (e.g., the label of class NCI_C12443 was changed from “Cortex” to “Cerebral Cortex”), were replaced by synonyms (e.g., the label of class NCI_C33178 was changed from “Nostril” to “External Nare”), or minor spelling or syntax modifications were inserted (e.g., the label of class MA_0000475 was changed from “aortic arch” to “arch of aorta”), while the meaning of the classes remained stable and the correspondences were still valid. However, the check also pointed us to six mistakes in the alignment that seem to have been caused by shifts in the mapping table. For example, the class NCI_C49334 “brain white matter” was mapped to MA_0000810 “brain grey matter” and NCI_C49333 “brain gray matter” to MA_0000820 “brain white matter”. For the LOD and the BRIDGE alignments we did not have access to URI-label pair data and so we could not run this check.

### Check 3

The ANATOMY and the BRIDGE reference alignments are distributed in the Alignment API format proposed by Euzenat and thus can easily be accessed and used via the associated JAVA-based Alignment API [14]. In contrast, the LOD alignments are not represented in a standard format but come in a comma delimited three column format. They contain typical mistakes often found in manually created documents, such as additional whitespace or (few) missing values, which, however, hinder automatic processing.

### Check 4

While classes in the ANATOMY and the BRIDGE alignments are referred to by class URIs, in the LOD data set local names are used to refer to classes. A major problem with the local names approach is that although local names are unique within a particular name space, across different name spaces they are generally not. Many ontologies mix up classes from different name spaces or even import whole ontologies with classes from a different name space. This almost inevitably leads to ambiguities (i.e., in case of an ambiguous local name in an alignment file, it is not clear to which ontology class the local name refers to). In all LOD alignments together we found 30 cases of ambiguous local names.

### Check 5

In the ANATOMY and the LOD data set, for each correspondence the relation holding between the two classes involved is explicitly specified. In contrast, regarding the BRIDGE data set, we encountered the problem that although all correspondences are marked as being based on the *equivalentClass* relation, according to a curator of the data set these specifications are rather a technical artifact, while, in fact, many of the correspondences are based on *subClassOf* and *relatedTo* relations (as mentioned before, the distinction between relation types had no relevance in the evaluation the data set has originally been created for). To cope with this situation, we decided to consider all relation types in the BRIDGE data set as being unknown (or more precisely, as being “one of *equivalentClass*, *subClassOf* or *relatedTo*”)*.* Unfortunately this also meant that we could not run the remaining checks on this data set, since they require type information.

### Check 6

We found 39 cases in the ANATOMY data set and 10 cases in the LOD data set in which a class from one input ontology was associated with more than one (target) class in the other input ontology of an alignment by an *equivalentClass* relation. In none of the cases, the target classes were linked by an *equivalentClass* relation in the respective input ontology themselves. We manually inspected all cases of multiple mapping targets. We found for the ANATOMY data set that in 20 cases, the target classes, in fact, seem to be equivalent classes that are just not yet marked appropriately in the given versions of the respective ontologies. Cross-checking with the most recent versions of the input ontologies revealed that from this set 12 target class pairs from the NCI meanwhile have been merged. For another three cases, we proposed a merger to the NCI team (for example, for the classes NCI_C33708 “suprarenal artery” and NCI_C52844 “adrenal artery”). Meanwhile they have been accepted and included in the new version release. Furthermore, we identified 18 cases in the ANATOMY alignment and five in the LOD alignments in which the target classes were linked by relations other than *equivalentClass* in the respective input ontologies. In twelve cases the target classes were linked by *partOf* relations (ANATOMY), in eight cases by *subClassOf* relations (four cases being from ANATOMY, another four from LOD), in two cases they were treated as sibling classes (ANATOMY), and in one case as disjoint classes (LOD). We inspected these relations and judged the majority of them as being correct. This allowed us to draw the conclusion that for the classes concerned only the mapping to one target class is correct, while the others should be removed from the alignment. The same, we found, applies for other five cases of multiple mapping targets in the LOD alignments, although in these cases no relations between the target classes were present in the respective input ontologies.

### Check 7

10,415 *subClassOf*-based correspondences could be inferred for the ANATOMY data set and 772 for the LOD data set simply by exploiting *equivalentClass*-based correspondences from the manual alignments in combination with the taxonomic structure of the corresponding input ontologies. While in the case of the ANATOMY data set all detected correspondences are new (because of its innate focus on *equivalentClass*-based correspondences), for the LOD data set we still found 70% (540) of the detected correspondences to be new (i.e., not contained in the alignments, yet).

### Check 8

After applying a simple term normalization procedure to all class labels (splitting of “Camel-Case” expressions, lowercasing, and underscore removal), for the ANATOMY data set we found 13 class pairs and for the LOD alignments a total of 37 class pairs (each consisting of a class from one and a class from the other input ontology) with identical labels for which no *equivalentClass*-based correspondence existed in the respective manual alignment. A manual inspection revealed that in the ANATOMY data set in two cases the respective classes, in fact, referred to slightly differently defined concepts. For example, the classes MA_0000323 and NCI_C12378 share the label “gastrointestinal system”. However, the MA class fits the usual understanding of “gastrointestinal system” comprising the stomach, intestine and the structures from mouth to anus, while the NCI class does not, but includes, in addition, accessory organs of digestion, such as the pancreas and the liver. (The NCI anatomy branch comes with another class, NCI_C22510 “gastrointestinal tract”, which corresponds to MA_0000323 “gastrointestinal system”). In the LOD data set we found three cases in which we think that, indeed, a *subClassOf* and not an *equivalentClass*-based correspondence can be stipulated for the class pair with a common name. For example, for a class pair sharing the name “Genre” one class seems to refer to the general notion of “genre”, while the other seems to be restricted to “music genre”. However, in the remaining 11 cases in the ANATOMY data set and 34 in the LOD data set *equivalentClass*-based correspondences are effectively missing in the respective alignments. An example is the class pair (NCI_C33460, MA_0002730) from ANATOMY sharing the label “renal papilla”. In the LOD data set some input ontology pairs import classes from the same third-party ontologies, e.g., the Time Ontology [15]. Thus, in nearly half of the analyzed cases it turned out that the classes did not only have identical names, but, in fact, denoted the same classes.

### Check 9

After class label normalization (see above), we found 3,127 class pairs in the input ontologies of the ANATOMY alignment and 57 class pairs in input ontologies of the LOD alignments for which the following conditions applied: the label of one class included the label of the other one, both shared the same syntactic head, and no *subClassOf*-based correspondence for the class pair existed in the respective manual alignment. We manually analyzed the cases from the LOD data set and found that 52 *subClassOf*-based correspondences were, in fact, missing in the respective alignments, of which 24 had already been detected by Check 7. Five proposed *subClassOf* relations we judged to be imprecise or wrong. For example, for the classes named “Label” and “RecordLabel” we judged that in fact an *equivalentClass*-based correspondence should be added to the LOD alignments, instead of a *subClassOf*-based one and for the classes named “Book” and “InBook” and “Conference” and “Attending-A-Conference” no correspondence should be added at all.

### Check 10

We found that in the ANATOMY data set 916 correspondences (60%) and in the LOD data set 158 correspondences (7%) are trivial ones. In the BRIDGE data set we could not compute the number of trivial correspondences because of the missing relation type specification for the correspondences in the alignments.

Table 2 summarizes our observations.

**Table 2:** Results of applying the ten basic quality checks to the Anatomy, Lod and Bridge data sets.

Check	Description	ANATOMY	LOD	BRIDGE
**Check 1**	*Correct input given?*	No (other versions)	No (only URLs)	No (only URLs)
**Check 2a)**	*Missing classes*	0	143 (47)	12
**Check 2b)**	*URI-label pair changes*	121	Not applicable	Not applicable
**Check 3**	*Standard format?*	Yes	No	Yes
**Check 4**	*URIs?*	Yes	No (local names)	Yes
**Check 5**	*Explicit relation types?*	Yes	Yes	No (untyped)
**Check 6**	*Multiple targets*	39	10	Unknown
**Check 7**	*New* ⊆ *inferred from* ≡	10,415	540	Unknown
**Check 8**	*Label identity but no* ≡	13	37	Unknown
**Check 9**	*Label inclusion but no* ⊆	3,127	57	Unknown
**Check 10**	*Trivial correspondences*	916 (60%)	158 (7%)	Unknown

Results of applying the ten basic quality checks to the ANATOMY, LOD and BRIDGE data sets. The symbols ≡ and ⊆ stand for *equivalentClass* and *subClassOf*-based correspondences, respectively.

## Discussion

Checks 1 to 5 mainly address technical issues (data availability and format requirements, etc.). They revealed that the available reference alignments are usually distributed without the original input ontologies they are based on. Additionally, various distributions suffer from shortcomings which typically occur in manually created documents for later use in automatic processing pipelines (coding errors, inconsistent formats, naming errors, etc.).

Before we were able to run the checks on the LOD and the BRIDGE data sets at all, we had to carry out extensive and time-consuming preparatory work. This dilemma arose for the input ontologies (search the Web for missing ontologies, solve recursive ontology import problems, etc.), as well as for the alignments themselves. The alignments, in particular, posed all sorts of problems. For example, the use of non-standard formats containing certain irregularities hampered automatic processing. Furthermore, the allowance for ontology elements other than those of type rdfs:Class or owl:Class as constituents of correspondences often caused problems depending on the tool employed for processing the alignments. Finally, the use of ambiguous local names to refer to classes, instead of unique identifiers, as well as spelling mistakes and name space confusions in the alignment files hindered the automatic lookup of classes from the alignment in the input ontologies. Interestingly enough, we encountered problems with spelling mistakes not only in alignments represented in non-standard formats (although they contained considerably more mistakes), but also in those available in a machine-processable format. A simple reason for this might be that a manually typed list of class names or URIs was used as input for the automatic creation of the final alignment files. For manually created documents (ontology files, mapping tables, etc.) that undergo a lot of copy-and-paste activities and for which proper spelling as well as case-sensitivity and special delimiters are crucial for distinctive naming, we thus recommend automatic forms of sanity-checking and data cleansing.

On top of that, it was simply disappointing that the very promising BRIDGE data set (which looks impressive both in terms of its domain coverage and the number of correspondences it contains) comes without any relation type encodings. This is even more deplorable because the creators of the alignments must have known the relation type holding for each single correspondence. According to the authors, the information was not kept, because it was not needed in the evaluation *at that time.*

Checks 6 to 9 target the content level of alignments. Much to our surprise, already simple procedures like searching for evidence of missing or erroneous correspondences in the alignment itself or in the respective input ontologies (exploiting structural and language features) turn out to be quite effective. Whether results from Check 7 are included in an alignment or not is rather a design choice that has to be justified by an alignment creator. We recommend either to consider all automatically inferable *subClassOf*-based correspondences in an alignment, or none of them. But we would definitely refrain from the inclusion of only some of them, a decision taken for the LOD data set. Certainly, the results from these checks can only be judged with caution since we cannot guarantee that we really worked with the ontology versions from the original settings as input (see the results of Check 1).

When we applied the quality checks to the ANATOMY, LOD and BRIDGE data sets the individual strengths and weaknesses of each data set became apparent. For the LOD data set we could derive suggestions for improvement from all ten checks. For the LOD and the BRIDGE data sets the format checks proved to be particularly helpful for clarifying how the usability of the data set could be substantially improved. In contrast, the ANATOMY data set has been used in a public evaluation campaign for several years now and possible technical obstacles have already been removed. For this data set, the checks at the content level, in particular, rendered very positive effects.

By far the most interesting results were achieved analyzing the outcomes of Check 2b), 6, and 8 for the ANATOMY data set. These checks helped us detect a total of 30 erroneous correspondences that needed to be removed from the reference alignment (this accounts for 2% of the complete alignment and 5% of its non-trivial subset) and 14 new ones that we proposed to add to the alignment. The list of invalid and newly proposed correspondences was communicated to the anatomy alignment curators and the organizers of the OAEI Anatomy track, and meanwhile has been incorporated in the OAEI 2011 campaign.

In addition, the results of Checks 7 and 9 can be taken as a “first aid” for a possible effort to extend the alignment to *subClassOf*-based correspondences. Finally, Check 10 revealed that only one third of the correspondences in the ANATOMY alignment are non-trivial, i.e., they cannot be detected by simple string matching tools. Since the alignment is quite large with respect to the number of correspondences, this makes it still a valuable evaluation data set. However, the large percentage of trivial correspondences must be considered by evaluation metrics when interpreting the results alignment systems achieve on this data set, or when comparing these results to those achieved by the same systems on different data sets. (The OAEI Anatomy track organizers are aware of this fact and compute, in addition to standard recall and precision, a measure they call “recall+”. It refers to the non-trivial correspondences a system is able to detect.)

For the LOD data set, the analysis of the results of Checks 6, 8 and 9 revealed that at least 10 erroneous *equivalentClass*-based correspondences exist in the LOD alignments that should be removed (Check 6) and 35 new *equivalentClass*-based correspondences (34 from Check 8, one from Check 9) as well as 52 *subClassOf*-based correspondences should be added (Check 9). If, in addition, full results from Check 7 are considered, the number of newly proposed *subClassOf*-based correspondences is even higher.

An issue we did not focus on in our study relates to checking the logical consistency of an alignment. With regard to this challenging problem, we refer the reader to related work, e.g., by Meilicke *et al. *[16] who propose a Web-based tool that supports the human alignment curator in detecting and solving conflicts in an alignment by capitalizing on the outcome of logical reasoning processes.

We would like to emphasize that although we focused in this work on RDF(S)/OWL ontologies (i.e., we used the respective terminology and implemented the quality checks accordingly) the basic idea behind each proposed quality check is independent from the representation format being used.

## Related work

Apart from very few exceptions (such as the work by Ceusters, introducing a metric for measuring the quality of both, the input ontologies of an alignment and the ontology resulting from it [17]), in the literature, alignment quality is primarily discussed in the light of the evaluation of *automatically* created alignments. Different approaches have been proposed to evaluate the performance of automatic alignment systems and the output they produce. These include the manual analysis of correspondences in the alignment [7], comparing the alignment against a reference alignment [4,5], measuring the extent to which the alignment preserves the structural properties of the input ontologies [18], checking the coherence of the alignment with respect to the input ontologies [19], and evaluating the alignment within an application (end-to-end evaluation) [20], while the comparison against (preferably manually created) reference alignments is by far the most common evaluation approach that has been used in international evaluation campaigns for many years now [21]. However, although the manual creation of ontology alignments is known to be time consuming and expensive, and in the same time inherently error-prone (Euzenat even states that “*humans are not usually very good at matching ontologies manually*”, p. 202 in [1]), not much work has been published on quality assurance of existing *manual* alignments, yet. This particularly concerns technical aspects, which strongly affect the reusability of alignments (target of our Checks 1 to 5). Regarding validity aspects (target of our Checks 6 to 10), some of the evaluation approaches proposed for the analysis of automatically created alignments could be adopted, such as the structural [18] or alignment coherence analysis [19].

## Conclusions

We presented ten basic quality requirements and associated checks intended to assist developers and curators of ontology alignments to create and maintain both, valid and easy to (re)use references for the evaluation of alignment systems. As we could show – using the example of the OAEI Anatomy track reference alignment and two additional reference data sets – very basic checks are already of great help to increase the quality of alignments. While checks addressing technical issues such as data availability and format requirements have proven to be important for increasing the *usability* of manual alignments, checks on the content level have shown they can foster its usefulness and *validity* through the detection of missing correspondences that should be added to an alignment and incorrect correspondences that should be removed from it. We also observed that the tests can reveal shortcomings in the input ontologies themselves, such as missing or invalid relations between classes.

The set of basic checks we formulated in this article should be considered as a first, rather simple, yet effective step in a multi-stage procedure of extensively checking the quality of an alignment before it is used as a reference in an evaluation setting. Our work is thus targeted at the sanity of comparison standards, an issue of prime importance for any conclusion we can draw from the outcome of any evaluation campaign. We propose to complement the basic checks by more advanced logical consistency checks and more elaborate considerations on alignment quality as described, e.g., by Joslyn *et al. *[18] who check for the structural preservation of semantic hierarchy alignments.

## Competing interests

The authors declare that they have no competing interests.

## Author’s contributions

EB worked out the quality checks, ran them against the reference alignment data sets and analyzed the results. UH was involved in this research as EB’s doctoral adviser monitoring the design of the study. Both authors drafted the manuscript and read and approved the final version.
